# Atomic Force Microscopy: In Sickness and in Health

**DOI:** 10.1155/2019/6149247

**Published:** 2019-05-14

**Authors:** Stylianos-Vasileios Kontomaris, Colin Grant, Eleni Alexandratou, Andreas Stylianou

**Affiliations:** ^1^Mobile Radio Communications Laboratory, School of Electrical and Computer Engineering, National Technical University of Athens, Iroon Polytechniou, Athens 15780, Greece; ^2^Athens Metropolitan College, Sorou 74, Marousi 15125, Greece; ^3^Hitachi High-Technologies Europe, Techspace One, Keckwick Lane, Warrington WA4 4AB, UK; ^4^Biomedical Optics and Applied Biophysics Laboratory, School of Electrical and Computer Engineering, National Technical University of Athens, Iroon Polytechniou, Athens 15780, Greece; ^5^Cancer Biophysics Laboratory, Department of Mechanical and Manufacturing Engineering, University of Cyprus, Nicosia 2238, Cyprus

During the last decades, Atomic Force Microscopy (AFM) has become a powerful tool able to provide quantitative and qualitative information regarding many pathological issues, based on the determination of proteins', cells', and tissues' mechanical and topographical properties at the nanoscale. This special issue is aimed at exhibiting the latest research achievements, findings, and ideas in the field of AFM research regarding biological materials and pathological conditions. In order to provide a clear introduction and guideline to new researchers into this field, A. Stylianou et al. (2019) offered an overview regarding the basic principles of AFM and its applications in biology and medicine. The imaging and nanomechanical characterization abilities were briefly presented followed by a complete presentation of applications related to pathological conditions such as osteoarthritis, Alzheimer's disease, and cancer. In addition, the ability to provide useful information regarding proteins and viruses was discussed. Among the original researches reported in this special issue, Z. Chang et al. (2019) explored the nanoscale elastic modulus of the tunica media of the internal mammary artery (IMA) in hydrated and dehydrated conditions from the patients with low and high arterial stiffening, as assessed in vivo by carotid-femoral pulse wave velocity (PWV). Their research was conducted using the AFM PeakForce quantitative nanomechanical mapping (QNM) technique and revealed the utility of AFM methods for arterial stiffening studies.

In addition, E. Kozlova et al. (2019a) used AFM techniques in order to test the ability of membranes of native human red blood cells (RBCs) to bend into the cell to a depth comparable in size with physiological deformations. The significance of the aforementioned investigation is crucial since the results of the work can be used in clinical practice, in assessing the quality of stored donor blood for transfusion, in biophysical studies of RBC properties. Also, E. Kozlova et al. (2019b) used AFM to study the nanostructure of the spectrin matrix of RBCs in response to temperature changes. Their results can be used as the basis for understanding how an increase in body temperature can affect RBC membrane nanostructure, morphology, and, ultimately, blood rheology.

Furthermore, a significant contribution regarding the applications of AFM in the drug industry was performed by Z. Xu et al. (2019) who contributed towards the clarification of the mechanism of action between Fe_3_O_4_ NPs and biological membranes. Their contribution could probably have a potential application in designing the targeted drug liposomes.

In conclusion, the objectives of the special issue have been reached in terms of advancing the current state of the art of AFM applications in sickness and in health. Several basic problems in these areas were well addressed, and most of the proposed contributions exhibited very promising results that outperform existing studies in the community.

